# Molecular Mechanism of Functional Ingredients in Barley to Combat Human Chronic Diseases

**DOI:** 10.1155/2020/3836172

**Published:** 2020-03-30

**Authors:** Yawen Zeng, Xiaoying Pu, Juan Du, Xiaomeng Yang, Xia Li, Md. Siddikun Nabi Mandal, Tao Yang, Jiazhen Yang

**Affiliations:** ^1^Biotechnology and Germplasm Resources Institute, Yunnan Academy of Agricultural Sciences/Agricultural Biotechnology Key Laboratory of Yunnan Province, Kunming 650205, China; ^2^Bangladesh Wheat and Maize Research Institute, Nashipur, Dinajpur 5200, Bangladesh; ^3^Key Laboratory of the Southwestern Crop Gene Resources and Germplasm Innovation, Ministry of Agriculture, Kunming 650205, China

## Abstract

Barley plays an important role in health and civilization of human migration from Africa to Asia, later to Eurasia. We demonstrated the systematic mechanism of functional ingredients in barley to combat chronic diseases, based on PubMed, CNKI, and ISI Web of Science databases from 2004 to 2020. Barley and its extracts are rich in 30 ingredients to combat more than 20 chronic diseases, which include the 14 similar and 9 different chronic diseases between grains and grass, due to the major molecular mechanism of six functional ingredients of barley grass (GABA, flavonoids, SOD, K-Ca, vitamins, and tryptophan) and grains (*β*-glucans, polyphenols, arabinoxylan, phytosterols, tocols, and resistant starch). The antioxidant activity of barley grass and grain has the same and different functional components. These results support findings that barley grain and its grass are the best functional food, promoting ancient Babylonian and Egyptian civilizations, and further show the depending functional ingredients for diet from Pliocene hominids in Africa and Neanderthals in Europe to modern humans in the world. This review paper not only reveals the formation and action mechanism of barley diet overcoming human chronic diseases, but also provides scientific basis for the development of health products and drugs for the prevention and treatment of human chronic diseases.

## 1. Introduction

Global cost of five chronic diseases (diabetes, cardiovascular disease, mental illness, chronic respiratory disease, and cancer) treatment to reach $47 trillion from 2011 to 2030 [1]. The intake of high sodium with low whole grains and fruits was the top most dietary risk factors for deaths and disability-adjusted life years globally and in many countries [2]. Diabetes in 11 production regions of polished rice with high glycemic index (GI ≥ 70) caused the biggest reduction in health-adjusted life expectancy at birth in 21 regions in 187 countries from 1990 to 2013 [3]. The micronutrients deficiencies at the highest risk are Fe, Zn, and vitamins (V_B1_, V_B2_, V_B12_, and V_C_) [4]. The outbreak of human chronic disease is due to taste pursuit that changes a healthy diet, i.e., the ancients switched from brown rice (GI ≤ 55, high K and high micronutrients) and barley (GI ≤ 25) or its grass flour (K/Na ≥ 10) as staple foods to modern polished rice (GI ≥ 87) and wheat white flour (GI ≥ 86) with low and low micronutrients as staple foods [3, 5].

**Figure 1:**
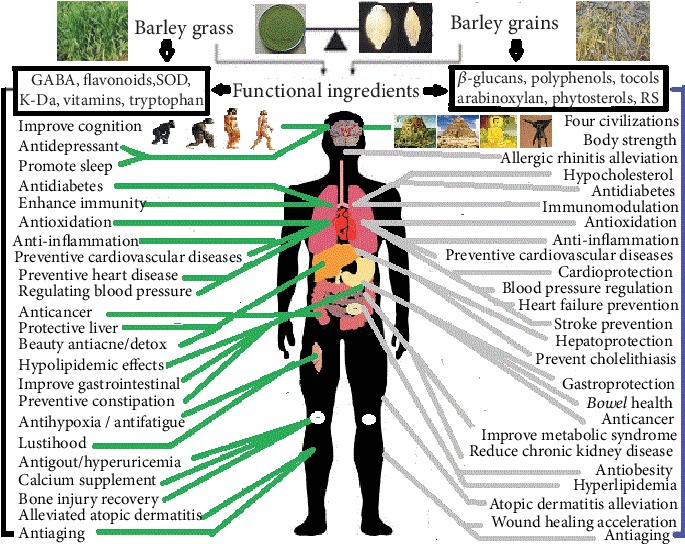
Barley grass and grains for preventive over 20 human chronic diseases.

Barley grass is not only the best functional food for cell nutrition and detoxification in human body but also the most abundant bioactive ingredients for lots of health-promoting effects [6, 7]. It can combat more than 20 chronic diseases due to GABA, flavonoids, SOD, K-Ca, vitamins, and tryptophan mechanism in barley grass (Figure 1) [6]. The sustaining major foods+barley grass powder can achieve the WHO's intake target of low sodium (<2 g) with high potassium (>3.5 g) every day [8]. More than 30 functional ingredients in barley grass can combat over 20 chronic diseases, and 15 functional ingredients in barley grains may prevent 11 chronic diseases [9]. Barley enhanced the sterols accumulation through *LTP2* gene action that take part in the abiotic stress reaction of mediating intracellular lipid transport [10]. Barley straw (2.0~8.0 g/L) for phenolic acid in degradation inhibited the alga (*M. aeruginosa*) blooms of aquatic eutrophication by cell shrinkage of metabolic activity and Chlorophyll a fluorescence decay [11, 12]. Therefore, barley grass powder plays an important role for solving human chronic diseases.

Barley grains have the highest functional value (low GI with high *β*-glucans and resistant starch) and antioxidant properties among cereal crops. The soluble fiber *β*-glucans is a group of polysaccharides found in barley, oats, mushrooms, yeasts, and seaweed [13]. Hulless barley variety Zangqing 320 has a 4.84-Gb sequence with 46,787 genes in seven chromosomes [14]; three *HvCslF* genes take part in (1,3; 1,4)-*β*-glucan synthesis [15]. Qingke (hulless barley) is a major food for Tibetan people and an important livestock feed in the Qinghai-Tibetan Plateau, which has lots of gene family related to stress reactions [16], especially different antioxidant capacities due to some polysaccharide and phytochemical compositions [17]. Regular daily consumption of whole barley flour can prevent chronic diseases, especially diabetes, colonic cancer, hyperlipidemia, high blood pressure, and gallstones [18]. Although barley grains have played an important role in health effects of human being, health contribution and different major mechanisms from barley grass for preventive human chronic diseases and functional ingredients in barley grains are unclear.

## 2. Functional Ingredients in Barley

Barley not only is a major feed and malt as well as a major food in some nations of the world but also is the richest grain source of functional ingredient and the most abundant species for functional food crop. Barley grains are extremely rich in nutritional functional ingredients (Table 1). Barley whole grains and its outer bran layer are rich in functional ingredients, especially fiber, phenolic acids, flavonoids, phytosterols, alkylresorcinols, benzoxazinoids, lignans, tocol, and folate, which have antidiabetes, anticancer, antiobesity, preventive cardiovascular disease, antioxidant, antiproliferative, and cholesterol lowering abilities [18–20], such as *β*-glucan (2.40~7.42%) and total tocols (39.9~81.6 *μg*/g); 64 compounds in barley were 27 anthocyanins, 9 flavanols, 9 flavone glycosides, and 19 phenolic acids and aldehydes [21].

Green malt has the highest functional ingredients including antioxidant activity (79.80%), total phenolic (122.43 mg/100 g), (+)-catechin (69.06 mg/100 g), quercetin (30.78 mg/100 g), 1,2-dihydroxybenzene (37.21 mg/100 g), isorhamnetin (22.44 mg/100 g), and carotenoid (0.171 mg/100 g) [22]. The germ and the outer layers of hulless barley grains have the highest folate concentration (103.3 mg/100 g) [23]. The average concentrations of K, Ca, and Fe in barley grass powder were 6.7, 12.0, and 4.6 times that of barley grains, especially the *qK1/qMg1/qCa1* region with large additive effects in Bmag0211~GBMS0014 on chromosome 1H [24]. All the high-altitude (1,200~3,500 m) hulless barley can increase higher functional ingredient content (*β*-glucan 7.5-30.8%, arabinoxylan 39.8-68.6%, anthocyanin 11.0-60.9%, and metal chelating activity 16.6-43.2%) than that of plains (97~126 m altitude), but the soluble *β*-glucan and arabinoxylan content ranged from 2.0% to 2.8% and 0.08% to 0.19%, respectively [25].

### 2.1. *β*-Glucan

*β*-Glucan in barley is the most abundant group of polysaccharides in cell wall. The molecular weight of *β*-d-glucan in hulless barley grains is 571.4 kDa, which composes of (1 → 4)- and (1 → 3)-glucopyranosyl residues, especially its trisaccharide and tetrasaccharide accounted for 66.6% of total cellulosyl units [26]. *β*-Glucan content (%) in naked, malt, black, waxy-naked, and blue barley is 3.44, 3.46, 6.08, 6.75, and 5.91, respectively, especially waxy-naked barley flour has the highest extraction rate (95.49%); however, GI *in vitro* starch digestibility was lowered by adding *β*-glucan [27]. The fatty acid derived flavouring substance (dodecanoic acid, octyl butanoate, ethyl decanoate, and decyl acetate) in beer has important role in the aggregation behavior of barley *β*-glucan [28].

The *β*-glucan concentrations in the six hulless barley grains varied from 4.96% to 7.62%, among shorts (8.12~13.01%)>bran (6.15~7.58%)>flour (2.48~2.95%) [29]. The total *β*-glucan contents in the nine hulless barley are 4.7~6.3% in bran and >3.4~4.4% in refined flour [30].

### 2.2. Polyphenols

#### 2.2.1. Polyphenols in Hull Barley

Phenolic compounds have the antioxidant, anti-inflammatory, and antitumor potentials [31]. There are the most abundant polyphenols in barley grains, especially p-hydroxybenzoic (17.6%), p-coumaric (15.2%), and ferulic acids (54.4%) [32]. The most abundant polyphenol in barley extract include free polyphenols (98.0 ± 10.0 mg/100 g) and bound polyphenols (51.0 ± 2.0 mg/100 g), especially ferulic acid (27.77 mg/100 g) and >procyanidin B (7.37 mg/100 g) [33]. Phenolic acids such as ferulic acid and p-coumaric acid were 0.215 mg/100 g and 0.110 mg/100 g in barley grains and 0.407 mg/100 g and 0.144 mg/100 g in malt, respectively [34]. The extraction polyphenols yield of barley lactobacillus fermented solution of 60% ethanol concentration was 1.809%, main components (mg/100 g) including rutin (3.508), vanillic acid (2.128), ferulic acid (1.938), coumaric acid (1.136), gallic acid (0.680), protocatechuic acid (0.299), and p-coumaric acid (0.083) [35]. Compared to the raw barley extract, the protein, total phenols, and *β*-glucan of fermented barley extract with Lactobacillus plantarum dy-1 can significantly increase to 34.94%, 13.61 mg/g, and 13.44%, respectively [36]. There were larger genetic variations in the contents of total polyphenol (203.314 ± 34.256 mg/100 g), total flavonoid content (88.042 ± 14.343 mg/100 g), and antioxidant activity (41.55 ± 7.82%) among the 223 barley genotypes; however, major QTLs between bPb-0572 and bPb-4531 control phenolic compounds in Tibetan wild barley, especially the UDP-glycosyltransferase gene with biosynthesis of flavonoid glycosides was colocated with bPb-4531 [37].

#### 2.2.2. Polyphenols in Hulless Barley

NaCl stress increased the phenolic compounds (vanillic acid, p-coumaric acid, ferulic acid, and sinapic acid) accumulation and synthesis by upregulating the gene expression of phenylalanine ammonia lyase, cinnamic acid 4-hydroxylase, 4-coumarate coenzyme A ligase, p-coumaric acid 3-hdroxylase, and caffeic acid/5-hydroxyferulic acid O-methyltransferase of germinated hulless barley [38]. The blue hulless barley grains have larger variation on phenolic compounds and antioxidant activity, such as the free, bound, and total phenolic acids varied between 166.2~237.6, 170.1~240.8, and 336.3~453.9 mg/100 g, respectively, where the major phenolic compounds include quercetin, rutin, naringenin, hesperidin, (+)-catechin, gallic acid, benzoic acid, syringic acid, and 4-coumaric acid [39]. The anthocyanin and total phenolic contents in hulless barley grains are higher for high altitude, and the contents in its refined flours were 0.39~0.76 mg/100 g and 129.9~160.7 mg FAE/100 g and in its bran were 1.85~2.65 mg/100 g and 367.1~443.5 mg FAE/100 g, respectively [30]. Whole grain hulless barley had high contents of total phenolic (259.90 mg/100 g), total pentosan (10.74 g/100 g), and ORAC values (41.805 ± 0.565 mol/100 g) [40]. The bran extract of hulless barley rich in phenolic acids on the N*ε*-carboxymethyllysine formation during processing biscuits, which can reduce glycation and benefiting health [41]. Total polyphenols (291.7 mg/100 g) and proanthocyanidins (163.0 mg/100 g) as well as antioxidant capacities (1.45 mol/100 g) were the highest in the grains of barley RILs [42].

#### 2.2.3. Flavonoid

The largest group of natural polyphenols are the flavonoids [43]. Pigmentations play a protective role under stress conditions, which are caused by flavonoids (yellow, purple, and blue) of *Ant2* gene in anthocyanin biosynthesis in barley grain pericarp, meanwhile *Ant1* associated with gene encoding an R2R3 myeloblastosis transcription factor [44], but black is caused by phytomelanins and oxidized as well as polymerized phenolic compounds [45]. There are larger variation on free (20.61~25.59 mg/100 g), bound (14.91~ 22.38 mg/100 g), and total flavones (37.91~47.98 mg/100 g) in 12 blue hulless barley grains [39]. The *Blp* (black lemma and pericarp) locus with the synthesis of ferulic acid and other phenolic compounds in black barley revealed the increased antioxidant capacity on stress tolerance [46]. The flavanone-3-hydroxylase locus completely cosegregated with the barley *Ant17* position on the chromosome arm 2HL [47]. One dominant gene Blp1 for synthesized phytomelanin of the lemma and pericarp in black barley cosegregated with a 1.66 Mb between HZSNP34 and HZSNP36 on chromosome 1H [48].

#### 2.2.4. Anthocyanins

Anthocyanin for human health belongs to flavonoids, which is a secondary metabolite that plants adapt to harsh environments. *Ant2* gene (2HL) with a *bHLH* domain control purple grain and *Ant1* gene control red leaf sheath and pericarp in barley, *R2R3-MYB* (*Ant1*)+*bHLH* (*Ant2*) complex promotes the synthesis by affecting expression of the anthocyanin biosynthesis structural genes (*F3*′*h*) and *Ans* genes [49]. The 2HL alleles from barley purple pericarp synthesis the peonidin-3-glucoside [50]. The anthocyanin synthesis *HvMyc2* gene is the major variant factor for blue aleurone of barley [51]. Barley anthocyanins take part in the amino acid biosynthesis, carbon metabolism, phenylpropanoid biosynthesis, and metabolic pathways [52]. Flavonoid 3 ′- hydroxylase (*F3*′*H*) and flavonoid 3′, 5′-hydroxylase (*F3*′*5*′*H*)-coding genes take part in anthocyanin synthesis in barley [53]. The anthocyanin bran-rich fractions of yellow (158.7 mg/100 g, 9 anthocyanins) and purple barley (353.4 mg/100 g, 15 anthocyanins) are 6 times higher than that of the whole grain flours (21.0 and 57.3 mg/100 g), especially cyanidin 3-glucoside, delphinidin 3-glucoside, petunidin 3-glucoside, delphinidin 3-rutinoside, and cyanidin chloride [54].

### 2.3. Polysaccharide and Arabinoxylan

Arabinoxylan in barley is the second highest cell wall polysaccharide [55]. Arabinoxylan in barley plays an important role in quality traits of malt and beer product [56]; however, arabinoxylan arabinofuranohydrolase I can be used as novel enzyme products in the beer industry [57]. The starch degradation for seedling relies on cell wall degradation, where the iminosugar 1,4-dideoxy-1,4-imino-l-arabinitol inhibits dextrinase and arabinoxylan arabinofuranohydrolase but permits rapid diffusion of *α*- and *β*-amylase [58]. Arabinoxylan contents in barley grains range from 4.2% to 5.4% [59] where it ranged from 0.53 mg/100 g to 0.90 mg/100 g at an average value of 0.67 mg/100 g in barley endosperm in 128 spring 2-row barley; its two QTLs include glycosyltransferases and glycoside hydrolases [55]. Arabinoxylan accounts for 45% of total polysaccharide [60] and 50~83% of total monosaccharide in barley husk [61]. Arabinoxylan contents in hulless barley are in bran 8.42%>in shorts 4.08%>in flour 2.13% [29] which differ from the report of Moza et al. [30] (in bran 1.97~3.6%>in flour 0.7~1.1%).

### 2.4. Phytosterols

Phytosterols in plant membrane are similar in structure to cholesterol [62]. Higher phytosterols are found in the outer layers of barley grains and ranged between 82.0 mg/100 g and 115.3 mg/100 g, among which *β*-sitosterol is 47.6 ± 0.1 mg/100 g and campesterol is 18.1 ± 0.2 mg/100 g. The other phytosterols include stigmasterol (3.9 mg/100 g), brassicasterol, *δ*5-avenasterol, stigmastanol, stigmastadienol, and other minor sterols (*δ*5- and *δ*7-avenasterols, *δ*7-stigmastenol, and stigmastadienol: 8.6 ± 0.1 mg/100 g) [18].

### 2.5. Tocols

Vitamin E is the major lipid-soluble antioxidant for human health, which has eight different stereoisomers [63, 64] by three chiral centers in tocopherols from barley. Spring barley has higher *α*-tocotrienol content in four tocols (*β*-tocotrienol, *α*-tocotrienol, *β*-tocopherol, and *α*-tocopherol) [65]. Vitamin E in barley ranged from 0.850 to 3.15 mg/100 g dry weight meanwhile ascorbic acid equivalent antioxidant capacity varied from 57.2 to 158.1 mg/100 g fresh weight [66]. Tocotrienols and tocopherols have antioxidant activity for human health, organic cultivation can increase *α*-tocotrienol (3.05-37.14%) and (*β* + *γ*)-tocotrienol (15.51-41.09%) as well as *δ*-tocotrienol (30.45-196.61%), but decrease *α*-tocopherol (5.90-36.34%) and (*β* + *γ*)-tocopherol (2.84-46.49%) concentrations in barley [67]. A tocochromanol in barley grains ranged from 162.0 to 185.2 mg/100 g, but which is much higher than in oat (4.5 mg/100 g) and Triticum (107.0 mg/100 g) [68]. Tocochromanols content in barley is 50% in pericarp, >37% in endosperm, and >13% in germ; about 85% of the tocochromanols were tocotrienols, and tocopherols in germ (80%) was higher than that in pericarp (20%) [69].

The hulless barley especially with waxy, double waxy and Tercel cultivars have the highest tocols content. Tocol in whole grain was 5.38 mg/100 g to 12.49 mg/100 g, and in pearling flour was 19.5 mg/100 g to 36.3 mg/100 g; however, the ratios of total tocotrienols to total tocopherols ranged from 1.6 to 3.9 [70]. The highest content of tocols (6.03~6.76 mg/100 g) and vitamin E concentrations (1.80~2.01 mg/100 g) was found in the waxy barley, especially in the hulless waxy Washonubet (tocols 6.76 mg/100 g and *α*-tocotrienols isomer 4.21 mg/100 g) [71].

### 2.6. Resistant Starch

Resistant starch (RS) can prevent diet-related chronic diseases such as diabetes and colon cancer. RS in hulless barley grains is related with B-type granules and the amylopectin F-III fraction; however, sequential rate of enzymatic hydrolysis in diets is waxy>normal>high amylose barley [72]. RS of 209 spring barley cultivars approved and popularized during the past 100 years in Europe, in which RS content ranged from <1% to >15% [73]. RS content of unprocessed grains of high-amylose, normal, and waxy barley is 24.0 ± 0.8%, 17.0 ± 0.0%, and 9.2 ± 0.7%, respectively, but slowly digestible starch in unprocessed grains of normal (41.6 ± 0.1%)>high-amylose (23.5 ± 0.5%)>waxy barley (20.8 ± 0.2%) and rapidly digestible starch in normal (6.6 ± 0.1%)<high-amylose (10.7 ± 0.4%)<waxy barley (16.3 ± 0.5%) [74]. The RS content (mg/100 g) of 629 accessions barley grains was 1.56 ± 1.22%, with a highest content up to 9.0% [75].

SNPs (i.e., 10th exon G(3935)-to-T and fifth exon C(2453)-to-T) in three exons play different roles on the expression of the waxy transcript, granule-bound starch synthase I (*GBSS I*), protein, amylose, and starch properties of hulless barley [76]. The chronology of RS contents in different barley diets is ground pearled barley (9.4%)>pearled barley flakes (8.1%)>whole pearled barley (7.4%)>cut barley (7.2%)>steamed rolled barley (5.7%)>malted barley (4.8%)>barley flake (3.8%), but digestible starch is cut barley (67.0%)>pearled barley flakes (65.7%)>whole pearled barley (64.8%)>barley flake (63.9%)>ground pearled barley (63.7%)>steamed rolled barley (61.9%)>malted barley (11.3%) [77].

### 2.7. GABA and Linoleic Acid

GABA increased *α*-amylase gene expression by treating barley aleurone with exogenous GABA, especially *α*-amylase activity began to rise after about 24 h and reached a peak at 48 h [78]. The GABA content (mg/100 g) in 629 accessions of barley grains is 8.00 ± 3.92 mg/100 g, the highest up to 30.67 8.00 ± 3.92 mg/100 g [75]. The GABA has a very important role in mediating NaCl stress phenolic compounds accumulation in germinated hulless barley [38]. Linoleic acid content increased from 51.74% to 56.56% and oil from 1.73 to 2.13%, while oleic content decreased from 19.94% to 15.62% and palmitic acid from 18.53% to 17.33% during barley malting process [23].

### 2.8. Phytases

Hydrolyze phytate in barley associated the bioavailable nutrient elements (P, Fe, and Zn), which exists as a single gene (*PAPhy_a*) in barley, but as two or three homeologous copies in wheat [79]. The improvement of *HvPAPhy*_a transformed barley showed phytase activity increases up to 110-fold in green leaves, 19-fold in grains, and 57-fold in dry straw [80].

## 3. Mechanism of Functional Ingredients in Barley Grains for Preventive Chronic Diseases

### 3.1. Healthy Effects of Functional Ingredients in Barley Grains

#### 3.1.1. Antidiabetic Properties

Diabetes is a chronic metabolic disease with high mortality rates; therefore, search for novel natural inhibitors has gained much attention [81]. Major antidiabetic elements in barley are *β*-glucan, phenolic compounds (phenolic acids and flavonoids), phytosterols, tocols, arabinoxylan, and resistant starch (Table 2). Oxidative stress not only leads to insulin resistance, impaired glucose tolerance, b-cell dysfunction, ultimately diabetes but also can treat diabetes and obesity by phytochemicals (phenolic acids, flavonoids, phytosterols, and tocols) in barley [18]. Chronic consumption of foods with high *β*-glucans in barley can improve insulin resistance and lower the postprandial glucose response and increase satiety [82]. The *β*-glucan in hulless barley reduced the insulin resistance, arterial sclerosis, serum glucose, and serum lipid in high-fat mouse [83]. High phenolic content (168.7 mg/100 g) and low rapidly digested starch (38.7%) make barley muffin to modulate glycemic response [84]. The hypoglycemic effect of ethanol extract polysaccharide from barley malt is better for decreased fasting plasma glucose of the diabetes mice than that of water extract [85]. The boiled barley kernels evening meal can facilitate glucose regulation, increase the release of glucagon-like peptide-1, and reduce energy intake and fasting serum free fatty acids, mediated through gut microbial fermentation of the indigestible carbohydrates [86]. The glycemic index (GI = 82.8) of all-wheat bread is higher than that (GI = 57.2) of 60% wheat+40% barley flour (6.0% *β*-glucan) [87]. GI for barley with 4.6% *β*-glucan and oat tempe are 30 and 63, respectively [88]. The hulless barley can reduce postprandial glucose and improved insulin sensitivity by amino acid and biogenic amine profiles [89].

#### 3.1.2. Antiobesity

Major antiobesity components in barley are *β*-glucan, resistant starch, polyphenols, dietary fiber, arabinoxylan, tocols, and phytosterols (Table 2). *β*-Glucan in barley significantly treats obesity that reduced low-density lipoprotein, total cholesterol, and serum p-cresyl sulfate levels and increased flow-mediated dilation [90, 91]. Barley *β*-glucan can prevent visceral fat (≥100 cm^2^) obesity and increase faecal scores, but decreased nutrient digestibility and antiobesity [92, 93]. RS and *β*-glucans as well as soluble arabinoxylan were utilized mainly in the caecum, especially RS shifted the utilization of other polysaccharides to more distal parts of the colon of pigs [94]. Obesity and insulin resistance associated with bile acid changes and lower dietary fiber (*β*-glucan) in barley diet [95]. The aqueous extract of fermented barley has antiobesity effects due to *β*-glucan and phenolic acids (vanillic acid and ferulic acid) [36].

*β*-Glucans in black and blue hulless barley for preventive obesity were very higher than that of white one, based on its molecular weights, particle sizes, viscosities, binding capacities (fat, cholesterol, and bile acid), and inhibiting activities on pancreatic lipase [96]. The polyphenols extracted in black hulless barley show notable decreases in total cholesterol, low-density lipoprotein cholesterol, and atherosclerosis, but significant increase in high-density lipoprotein cholesterol levels [97].

#### 3.1.3. Cardiovascular Disease Prevention

Barley *β*-glucan can reduce low-density lipoprotein cholesterol and non-high-density lipoprotein cholesterol as well as alters the gut microbiota for preventing cardiovascular disease (see Table 2) [98]. Oxidative stress and inflammation are two important factors of atherosclerosis, and polysaccharide extracts with antioxidation and anti-inflammation of hulless barley prevent cardiovascular diseases [17]. Some other functional components of barley have been associated with cardiovascular health, such as polyphenols, phytosterols, lignans, tocols, and folate [18].

#### 3.1.4. Anticancer Effect

Major anticancer elements in barley are *β*-glucan, phenolics, arabinoxylan, phytosterols, lignan, and resistant starch (Table 2). Functional ingredients of barley with antioxidative and immunomodulatory activities are associated with anticancer effects [18]. Barley with high dietary fiber (*β*-glucan) has an important role for the prevention of colon cancer and cardiovascular diseases [99]; low molecular weight *β*-d-glucan can enhance antioxidant and antiproliferative activities [100]. Aqueous extract of fermented barley can induce subcutaneous transplantation tumor apoptosis that can be used for a nutrient supplement in the treatment of human colon cancer [101].

*β*-Glucans in hulless barley has anticancer activities *in vitro*, but its anti-inflammatory activities increased as their molecular weights decreased [42]. The bound phenolics in dehulled hulless barley have excellent antioxidant and antiproliferative effects to human liver cancer cells [102]. A water soluble polysaccharide (glucose : xylose : arabinose : rhamnose = 8.82 : 1.92 : 1.50 : 1.00) from hulless barley can inhibit colon cancer, which induce HT-29 apoptosis through ROS-JNK and NF-*κ*B-regulated caspase pathways [103].

#### 3.1.5. Antioxidation

Antioxidants are compounds that remove reactive oxygen species from cells, which play a dual role in aggravating and preventing diseases [104]. Major antioxidants in barley are phenolic compounds (phenolic acids, flavonoids, and anthocyanin), tocols (vitamin E), polysaccharide (arabinoxylan), dietary fiber, and phytic acid (Table 2). Antioxidant effects of polyphenols in barley are flavanols>flavonols (quercetin)>hydroxycinnamic acids (ferulic, caffeic, and coumaric acids) [33]. Malt has higher phenolic content (sinapinic acid and epicatechin) than its barley grains, which plays a key role on antioxidant stability of beer [105]. The antioxidant activity for anthocyanin in barley bran was markedly higher than that of whole grains flour [54]. Five of the seven associations in barley were with markers near genes associated with the tocochromanol (vitamin E with the most powerful antioxidants) pathway [106]. Overexpression of homogentisate geranylgeranyl transferase for barley enhanced the tocotrienol levels (*δ*-, *β*-, and *γ*-tocotrienol 10-15%) and antioxidant capacity (radical scavenging activity 17-18%) in barley seeds [107].

The antioxidant effects of dietary fiber in hulless barley bran were associated with total phenolic concentration, which had the DPPH (1,1-diphenyl-2-picrylhydrazyl radical 2,2-diphenyl-1-(2,4,6-trinitrophenyl)-hydrazyl) radical-scavenging activity and ferric-reducing antioxidant power [108]. GABA induces the accumulation of proline and total phenolics and enhances the antioxidant system in germinated hulless barley under NaCl stress [38]. The chapatti quality score reduced by 15% and its phenolic concentration increased from 23.7 to 28.7 mg/100 g, while biscuit spread factor reduced by 33% and its *β*-glucan concentration increased from 0.60 to 2.4% as well as phenolic content increased from 6.3 to 13.5 mg/100 g after blending of 30% hulless barley flour, especially markedly increased antioxidant activity [109].

#### 3.1.6. Anti-inflammation

Major anti-inflammatory ingredients in barley are *β*-glucans, lignans, vanillic acid, arabinoxylan, and so on (Table 2). Endothelial cell adhesion molecules were identified as an early step in inflammation and atherogenesis; barley *β*-glucans not only have a maximum anti-inflammatory activity at Mw~1.40 × 10^5^, especially the inhibition of TNF-*α*-induced expression of vascular cell adhesion molecule was stronger than that of oat *β*-glucans, but also have a higher ratio of 3-O-*β*-cellobiosyl-d-glucose to 3-O-*β*-cellotriosyl-d-glucose oligomers in the polymeric chains [110]. The molecular weights of *β*-glucans in hulless barley increased that add inhibitory abilities on *α*-amylase and pancreatic lipase, but the anti-inflammatory abilities decreased, especially the low intrinsic viscosity and high solubility of *β*-glucans acid hydrolysis for 20 min might contribute to its higher anti-inflammatory activity, which significantly affected their bioactivities (e.g., anticancer), which was beneficial for a better understanding of their structure-function relationships [111]. The fermented barley extracts (vanillic acid) downregulate glucose consumption and reducing proinflammatory cytokine secretion [112]. The anti-inflammatory property of malt and whole-grain barley is due to the formation of short chain fatty acid (SCFA) and changes in microbiota composition [113].

#### 3.1.7. Immunomodulation

Major immunomodulatory substances in barley are *β*-glucans, arabinoxylan, and so on (Table 2). The immunomodulatory activity of barley *β*-glucans insolubility associated with its particle size, granule conformation, and particulate homogeneity. All *β*-glucan fractions can induce more cytokines in bone marrow-derived dendritic cells than their oat equivalents; however, the insolubility of *β*-glucan affects its immunomodulatory activity, which is related to its particle size, particle configuration, and particle uniformity [114]. A water-soluble polysaccharide (BP-1, molecular weight 6.7 × 10^4^ Da) from hulless barley can improve the immune ability of immunosuppressive mice through increasing the serum levels of IL-2, TNF-*α*, and IFN-*γ*, such as BP-1 (80 mg/kg and 160 mg/kg) can not only significantly increase the number of bone marrow cells and peripheral blood white blood cells, as well as enhance the production of IL-2, TNF-*α*, IFN-*γ*, IgG, and IgM in the spleen and serum levels for improving the immune function, but also promote the proliferation and phagocytosis activity of macrophages as well as repair the damage induced by CTX in the spleen cells of immunosuppressive mice [115].

#### 3.1.8. Cardioprotection

Barley (1–3) *β*-d-glucan confers postischemic cardioprotection (see Table 2), which shows a 109% survival rate after cardiac ischemia (30 min)/reperfusion (60 min) injury, reduces left ventricular anion superoxide production (62%) and infarct size (35%), and increases the capillary (12%) and arteriolar density (18%) and VEGF expression (87.7%) of hearts in mice [116]. The products of barley grains can reduce the cardiometabolic risk and regulate the blood glucose and appetite hormones in 11-16 h after intake; however, its mechanisms are gut fermentation of indigestible carbohydrates [117].

#### 3.1.9. Hypocholesterolaemic effects

Cholesterol is a synthesis lipid in the body [118]. Dietary *β*-glucan of hulless barley reduces the plasma LDL cholesterol content (see Table 3) by promoting the excretion of faecal lipids and regulating the activities of 3-hydroxy-3-methyl glutaryl-coenzyme A reductase and cholesterol 7-*α*hydroxylase in hypercholesterolaemic rats [119]. Barley bran 5% and 10% in diet to the hypercholesterolaemic rats improved the level of lipids, lactate dehydrogenase, liver enzymes, and creatine kinase-MB [120]. Whole grain hulless barley has hypocholesterolaemic effects by promoting bile acid synthesis and reabsorption, controlling cholesterol synthesis and accumulation in peripheral tissue, decreasing the expression of 3-hydroxy-3-methylglutaryl coenzyme A reductase, while increasing the hepatic expressions of AMP-activated protein kinase *α*, cholesterol 7*α*-hydroxylase, LDL receptor, liver X receptor, and PPAR*α* [118].

#### 3.1.10. Blood Pressure Regulation

Higher consumption of barley *β*-glucan is associated with lower systolic and diastolic blood pressure (see Table 2), i.e., diets rich in *β*-glucans reduce systolic blood pressure by 2.9  mmHg (95% CI 0.9 to 4.9  mmHg) and diastolic blood pressure by 1.5  mmHg (95% CI 0.2 to 2.7  mmHg) for a median difference in *β*-glucans of 4  g [121]. The consumption of high molecular weight barley *β*-glucan can reduce blood pressure [98].

#### 3.1.11. Bowel Health Improvement

Gastrointestinal tract disease is a major global health problem. *β*-glucan in hulless barley has protective effects to the gastrointestinal tract (see Table 2) [122]. The dietary fiber in hulless barley improved indices of bowel health compared with refined cereal foods, especially Himalaya 292 possesses high amylose and resistant starch due to lacking activity of a key enzyme responsible for starch synthesis; however, consumption of Himalaya 292 foods resulted in 33% higher faecal weight, a lowering of faecal pH from 7.24 to 6.98, a 42% higher faecal concentration, a 91% higher excretion of butyrate, a 57% higher faecal total SCFA excretion, and a 33% lower faecal p-cresol concentration [123]. Fermented barley extract (10 mg/100 g) can act as a promising laxative agent to cure spastic constipation [124]. Butyric acid for improving the colonic health is produced by degradation of barley dietary fiber by microbiota [125].

#### 3.1.12. Gastroprotective effects

*β*-Glucan from hulless barley can mitigate the gastric lesions and gastric mucosal damage as well as gastric oxidative stress injury through decreasing the level of malondialdehyde [122]. The oral administration of fermented barley extract had strong gastroprotective effects through strengthening antioxidant defense system and anti-inflammatory effects, as well as decreasing lipid peroxidation and CAT activity by increasing the GSH levels and SOD activity in the body, and the 200 mg/kg dose of fermented barley extract was similar gastroprotective as the 10 mg/kg dose of omeprazole, which indicates that this dosage can be used for patients suffering from different levels of gastric damages [124].

#### 3.1.13. Reduce Chronic Kidney Disease

Barley *β*-glucans is associated with a saccharolytic shift in the gut microbiota metabolism by a reduction of pCS toxin blood levels and an increase of SCFA production at colonic site, which can reduce the microbial-derived uremic toxin and cardiovascular complications in end-stage renal disease (see Table 3), especially chronic kidney disease [90]. The total cholesterol and triglycerides were reduced, and HDL cholesterol increased in 10% and 20% barley intervention in breakfast diet; however, barley in the diet of stage 3 chronic kidney disease patients has significantly improved the nutritional status and renal functions [126].

#### 3.1.14. Improve Metabolic Syndrome

Barley *β*-glucan can improve postprandial glucose response and cholesterol levels as well as the metabolic syndrome based on individual gut microbiota composition (see Table 3) [127]. Tibetan hulless barley can reduce insulin resistance, dyslipidemia, and body weight gain, which can diminish the prevalence of metabolic syndrome induced by high-fat-sucrose diets, i.e., rats fed with Tibetan hulless barley can increase the assessment of insulin resistance scores (body weight, abdominal fat deposition, liver weight, liver fat deposition, triglyceride, fasting blood glucose, and serum fasting insulin) and decrease low-density lipoprotein cholesterol levels compared to rats fed with a basal diet [128].

#### 3.1.15. Hepatoprotective effect

*β*-Glucans in barley can decrease fatty liver in diabetes with obesity (see Table 2) [129]. The free phenolic extract in barley added the hepatic levels of antioxidant enzymes [130]. Whole grain hulless barley had significantly lower liver lipid levels (total phenolic and pentosan) [118]. Barley sprout extract protects liver cells under oxidative stress by activating Nrf2 and adding glutathione synthesis, especially against alcohol-induced liver injury, as it inhibits glutathione depletion and hepatic lipid accumulation, reduces serum biochemical markers of liver injury, and inhibits inflammatory responses [131].

#### 3.1.16. Wound Healing Acceleration

Nowadays, *β*-glucans represent effective topical agents for the treatment of chronic wounds and burns due to the activation of the immune and cutaneous cells (see Table 3), which increase wound repair by enhancing the infiltration of macrophages and promote tissue granulation, collagen deposition, and reepithelialization based on inducing the proliferation and migration of keratinocytes and fibroblasts through specific receptors such as Dectin-1, CR3, or TLRs [132]. Barley *β*-glucan in vivo promotes the wound closure in mouse skin by promoting the migration and proliferation of human dermis fibroblasts [133].

#### 3.1.17. Heart Failure Prevention

Barley *β*-d-glucan is a natural activator of MnSOD expression, which can prevent heart failure (see Table 3) [134]. The Food and Drug Administration has made a health claim between *β*-glucan and reduced risk of coronary heart disease, diabetes, and heart-related problems [135]. Barley products can prevent and reduce the risk of coronary heart disease, which is associated with the constituents like *β*-glucan, phenolics, tocols, linoleic acid, and folate [18, 136].

#### 3.1.18. Atopic Dermatitis Alleviation

The allergen produced by barley and the protein expressed in insect cells induce the same amount of IFN-*γ* and IL-4 in PBMC from vaccinated horses (see Table 2) [137]. Fermented barley extract P reduced skin lesions by inhibiting inflammatory cytokines [138], but GABA alleviated atopic dermatitis by suppressing serum immunoglobulin E and splenocyte interleukin production [139].

#### 3.1.19. Stroke Prevention

Barley intake reduces the risk of total stroke by significantly increasing the medium and small particle sizes of high-density lipoprotein cholesterol (see Table 3) [140]. Increasing the expression of antioxidant genes in the liver and the regulation of Nrf2 played a role in the regulation of metabolic diseases in stroke-prone spontaneously hypertensive rats consuming a fermented barley extract P diet [141].

#### 3.1.20. Allergic Rhinitis Alleviation

Ma-al-Shaeer formulation based on barley can treat for allergic rhinitis, especially reduced the nasal congestion, post nasal drip, and headache (see Table 3) [142]. Fermented barley extract alleviated allergic rhinitis in OVA-sensitized mice by regulating cytokines (IFN-*γ* or IL-17) related to chronic inflammation [138].

#### 3.1.21. Miscellaneous Disease Prevention

In addition, the common edible whole-barley flour can reduce the risk of hyperlipidemia and cholelithiasis [18], antiaging [140] as well as increase body strength and increase the content of linoleic acid and linolenic acid in pigs, cattle, sheep, and geese.

In a word, there are more than 20 kinds health effects for barley grain preventive chronic diseases according to the summary of current retrieval literature. The descriptions of some literatures for barley preventive chronic diseases (antidiabetes, antiobesity, anticancer, antioxidation, anti-inflammation, hypocholesterolaemic effects, blood pressure regulation, cardioprotection, immunomodulation, improve gastrointestinal, hepatoprotection, bowel health, cardiovascular disease prevention, atopic dermatitis alleviation, wound healing acceleration, heart failure prevention, and so on) are relatively sufficient, but some literatures for barley preventive chronic diseases (antiaging, reduce cholelithiasis, and increase body strength) are relatively less. These results fully demonstrate the health contribution relationship between barley functional food and human chronic disease prevention. The efficacy of preventing chronic diseases is related to barley genotype and its location, composition, extraction, and compatibility, and with the in-depth study of barley grain in prevention and treatment of human chronic diseases, the novel mechanisms for the prevention of chronic diseases may be ascertained as well as its putative role, either at major or minor level, would be further validated.

### 3.2. Major Mechanisms of Functional Ingredients in Barley Grains for Preventive Chronic Disease

#### 3.2.1. *β*-Glucans mechanism

*β*-Glucans can be used as candidates for the medication in the treatment of human chronic diseases [133]. *β*-Glucans have many bioactivities including antidiabetes; anticancer; antiobesity; anti-inflammation; immunomodulation; cardioprotection; lower cholesterol and lower blood pressure; improve bowel health, gastroprotection, and hepatoprotection; reduce chronic kidney disease and metabolic syndrome; prevent the risk of heart and cardiovascular diseases; and accelerate wound healing activities (Figure 1, Tables 2 and 3) [13, 18, 98, 111, 116, 122, 123, 127, 133, 143–149]. The major mechanisms of *β*-glucans of barley involve in the prevention of chronic diseases are as follows: *β*-glucans can interact with intestinal lipids and bile salt to reduce cholesterol levels and subsequently prevent diabetes, hypertension, cardiovascular disease, and metabolic syndrome [143]. Barley *β*-glucans not only control appetite and improve insulin sensitivity by gut hormone secretion via microbiota produced SCFA [144] due to its high molecular weight and high viscosity but also increase their antigen ability and enhances the proinflammatory cytokines, which can be degraded by macrophages and natural killer cells mediating its cellular cytotoxicity opsonized tumor cells [145]. *β*-Glucans of barley are the major regulators of adipogenesis, especially markedly downregulated the target genes in the adipose tissue including adipocyte fatty acid-binding protein, lipoprotein lipase, uncoupling protein-2, and glucose transporter 4 in 3T3-L1 cells [146] and also have the effect of inhibiting the *α*-amylase and pancreatic lipase [111].

Barley *β*-glucan can reduce blood pressure and cardiovascular diseases that alters the composition of gut microbiota, decrease body mass index, waist circumference, and triglyceride levels [98] and also reduce the systemic inflammatory profile, prevent alveolar bone loss, and improve *β*-cell function in diabetic animals [147]. Barley *β*-glucans can not only regulate immune responses and connect innate and adaptive immunity [13] but also have cardioprotective mechanism of promoted angiogenesis through endothelial upregulation of the vascular growth factor [116]. The hypocholesterolaemic effect of *β*-glucan in barley is due to the increased bile acid synthesis [148] and improved bowel health by inhibited feed intake and increased cecal fermentation [149]. Barley *β*-glucan not only has the gastroprotective effects by increasing the SOD and CAT activity, decreasing the gastric ulcer index, and increasing prostaglandin E2 and nitric oxide in laboratory rodents [122] but also affects lipid metabolism and SCFA production, lowering microbes in patients with metabolic syndrome [127] and improving chronic kidney disease by reducing the microbial-derived uremic toxin. Chronic consumption of barley *β*-glucans can decrease fatty liver by increasing small intestinal contents viscosity and improving glucose, lower glycated hemoglobin and relative kidney weights [129], strengthen the angiogenic ability of ROS-exposed endothelial cells for preventive heart disease [123], and accelerate the wound closure by promoting the migration and proliferation of human dermal fibroblasts [133].

#### 3.2.2. Polyphenols Mechanism

Barley polyphenols have lots of bioactivities including antidiabetes, antiobesity, anticancer, antioxidant, anti-inflammation, hepatoprotection, and prevention of cardiovascular and heart diseases (Figure 1, Tables 2 and 3). Barley lignan as natural polyphenols has anticancer, antioxidant, anti-inflammation properties, and preventive role for cardiovascular diseases. Anthocyanin belongs to flavonoids, and flavonoids belongs to polyphenols. Black, purple, and blue barley grains have gained much attention recently because their anthocyanins have anticancer, glycemic and body weight regulation, antioxidation, anti-inflammation, neuroprotection, hypolipidemia, retinal protection, hepatoprotection, and antiaging effects [150, 151].

Several mechanisms of barley polyphenols for preventing chronic diseases have been documented so far. Zhang et al. [36] has verified the molecular mechanism of insulin resistance by fermented barley extract vanillic acid through regulating miR-212 expression. The polyphenols with antiobesity from black hulless barley has strong superoxide radical, hydroxyl radical and 2,2-diphenyl-1-picrylhydrazyl radical-scavenging activity, ferric reducing antioxidant power, and moderate metal ion-chelating activity [97]. An efficacious antiproliferation capacity in Caco-2 cells of black barley malt free extract was predicted due to its phenolic constituents which have cellular antioxidant and oxygen radical absorbance as well as peroxyl radical scavenging activities, DPPH, and ABTS radical scavenging assays [152]. Black barley sprouting stimulates the phenolic biosynthesis by upregulating proline-associated pentose phosphate pathway to support structure of sprouts with antioxidant capacity [153]. Total phenolic and pentosan in hulless barley grains have antioxidant activity by downregulated expression of heat shock protein 60 and phosphatidylethanolamine binding protein 1 but upregulated expression of enoyl-coenzyme A hydratase and peroxiredoxin 6 [118]. The total polyphenol (flavonoid) content and the 2,2-diphenyl-1-picrylhydrazyl and ABTS radical scavenging abilities increased as the barley added to the food mixture [154]. Lignan (-)-7(S)-hydroxymatairesinol inhibited tumor necrosis factor-*α* stimulated endothelial inflammation by inhibiting NF-*κ*B activation and upregulating Nrf2 antioxidant element signaling pathway [155]. Lignans in barley have high anti-inflammatory abilities in endothelial cells by reducing nuclear factor-*κ*B and extracellular signal as well as regulating kinase phosphorylation [156]. The polyphenols such as (+)-catechin, protocatechuate, and quercetin in barley not only have hepatoprotective effect [130] but also prevent coronary heart disease by reducing oxidative-induced tissue damage through modulating intracellular signaling pathways [157]. Polyphenols play an important role in alleviating cardiovascular diseases due to their antiradical scavenging abilities [18].

#### 3.2.3. Arabinoxylan Mechanism

Barley arabinoxylan has a lot of health benefits, which include antidiabetes, antiobesity, anticancer, lowering cholesterol, immunomodulation, antioxidant, cardiovascular diseases prevention, and so on (Figure 1, Tables 2 and 3). Arabinoxylan in barley is the most abundant polysaccharide that has the capacity of lowering cholesterol and glucose as well as antioxidant activities [55]. The major mechanisms of barley arabinoxylan for preventing chronic diseases are as follows: arabinoxylan can improve 21 urinary metabolites associated with diabetes by improvement of carbohydrate and lipid as well as amino acid metabolism [158]. The antioxidant and antiobesity as well as immunomodulation of arabinoxylans associated with prebiotic effects and short-chain fatty acids production by interaction of gut microbiota and arabinoxylans [122]. Arabinoxylan rice bran can increase anticancer effects in the older population by increased NK activity [159]. The arabinoxylan in barley with the immunomodulatory activity consisted of a xylan backbone with acetate, arabinose, galactose, glucuronic acid, and 4-O-methylglucuronic acid [160]. The arabinoxylan diet led to a lower postprandial blood for glucose-dependent insulinotropic polypeptide response, especially fat oxidation has an important role in the antiobesity and in the prevention of cardiovascular diseases [161]. Barley polysaccharide prevent cardiovascular diseases by the vasodilatory effect of controlling angiotensin-converting enzyme production [17].

#### 3.2.4. Phytosterols Mechanism

Phytosterols are important micronutrients in human health. The outer layers in barley are the best source of plant sterols. Barley phytosterols have antidiabetes, antiobesity, and anticancer properties and can lower cholesterol and prevent cardiovascular diseases (Figure 1, Tables 2 and 3). Barley grains contain phytosterols that can esterify to fatty acids, phenolic acids, steryl glucosides, or acylated steryl glycosides [18]. Phytosterols significantly inhibited the ability of oxysterols to activate the liver X receptors transcription in modulating cancer cell behavior [62], which are thought to influence multiple processes related to cancer, such as carcinogen production, cancer-cell growth, angiogenesis, invasion, metasis, and cancer-cell apoptosis [162]. For the prevention of cardiovascular diseases, barley phytosterols can compete with cholesterol for micelle formation, inhibiting cholesterol absorption in intestine and lowering cholesterol in central nervous system of the brain [18, 162]. The effect of plant sterols on neurodegenerative diseases is due to its passage through the blood-brain barrier, modulating cholesterol metabolism and inflammation in the central nervous system process in the brain, which involve low-density-lipoprotein, apolipoprotein E, and scavenger receptor class B type 1 [162]. The lowering of cholesterol and prevention of cardiovascular disease are due to the fact that the phytosterol structure in the barley membrane is similar to the configuration of different cholesterol [18], but functionally similar to precursors in phytohormone synthesis, but lowering the blood concentration of cholesterol is beneficial to reduce the risk of cardiovascular disease.

#### 3.2.5. Tocols Mechanism

Tocol (Tocopherols and tocotrienols) is a class of lipid-soluble components found in barley, extremely rich in content in barley embryos. Barley tocols are the best in cereals due to a high concentration and favorable distribution of eight active vitamers [18]. Barley tocols have anticancer, antiobesity, and antioxidant effects and can lower cholesterol level, reduce the risk of stroke, and prevent cardiovascular and heart diseases (Figure 1, Tables 2 and 3) [18, 136]. Tocotrienols can suppress various cancers (breast, lung, ovary, prostate, liver, brain, colon, myeloma, and pancreas) by its molecular mechanisms of cellular proliferation, apoptosis, angiogenesis, metastasis, and inflammation [63], which are associated with human intake of whole grains, especially barley and rye. Tocotrienol is a functional food of obesity and diabetes by regulating adipogenesis and increase apoptosis of adipocytes and improve glucose homeostasis through suppression of inflammation and oxidative stress [64]. Tocol is one of the most powerful antioxidants that has the ability to interact with polyunsaturated acyl groups and scavenge lipid peroxyl radicals and quench reactive oxygen species, thus protecting fatty acids from lipid peroxidation [163]. All barley pitas had the greatest antioxidant and vitamin E levels from barley malt flour [66]. The antioxidant properties of barley tocols due to its ability to inhibit lipid peroxidation in biological membranes induce the immune system, promote apoptosis induction, and reduce the risk factors of cardiovascular diseases and stroke by atherosclerotic blockages in the carotid artery [18].

#### 3.2.6. Resistant Starch Mechanism

Barley RS has many immune properties like antidiabetes, antiobesity, anticancer, and so on (Figure 1,Table 2). Ceramide can promote lipid storage, impaired glucose utilization, and inhibited enzyme dihydroceramide desaturase 1, which can treat hepatic steatosis and metabolic disorders [164]. Antidiabetes of RS can be increased by suppressing amylopectin synthesis through silencing of starch branching enzymes in barley [165]. T cell development and gut IgA production suppress host lipid absorption by modulating CD36 expression [166] to achieve the effect of antiobesity of barley RS. GBSS I is mainly responsible for amylose synthesis whereas SSS I and SBE II for amylopectin synthesis in amyloplasts [167]. Barley with high *β*-glucan and moderate RS may benefit hyperglycemia-impaired lipid metabolism [168]. The blending of barley starch citrate with resistant starch IV up to 20% can produce noodles of acceptable quality and numerous health benefits [169].

## 4. Health Contribution and Preventive Role of Chronic Diseases of Barley

### 4.1. Formation Mechanism of Functional Ingredients Dependence

#### 4.1.1. Formation Mechanism of Depending Functional Ingredients for Early Hominids

**Figure 2:**
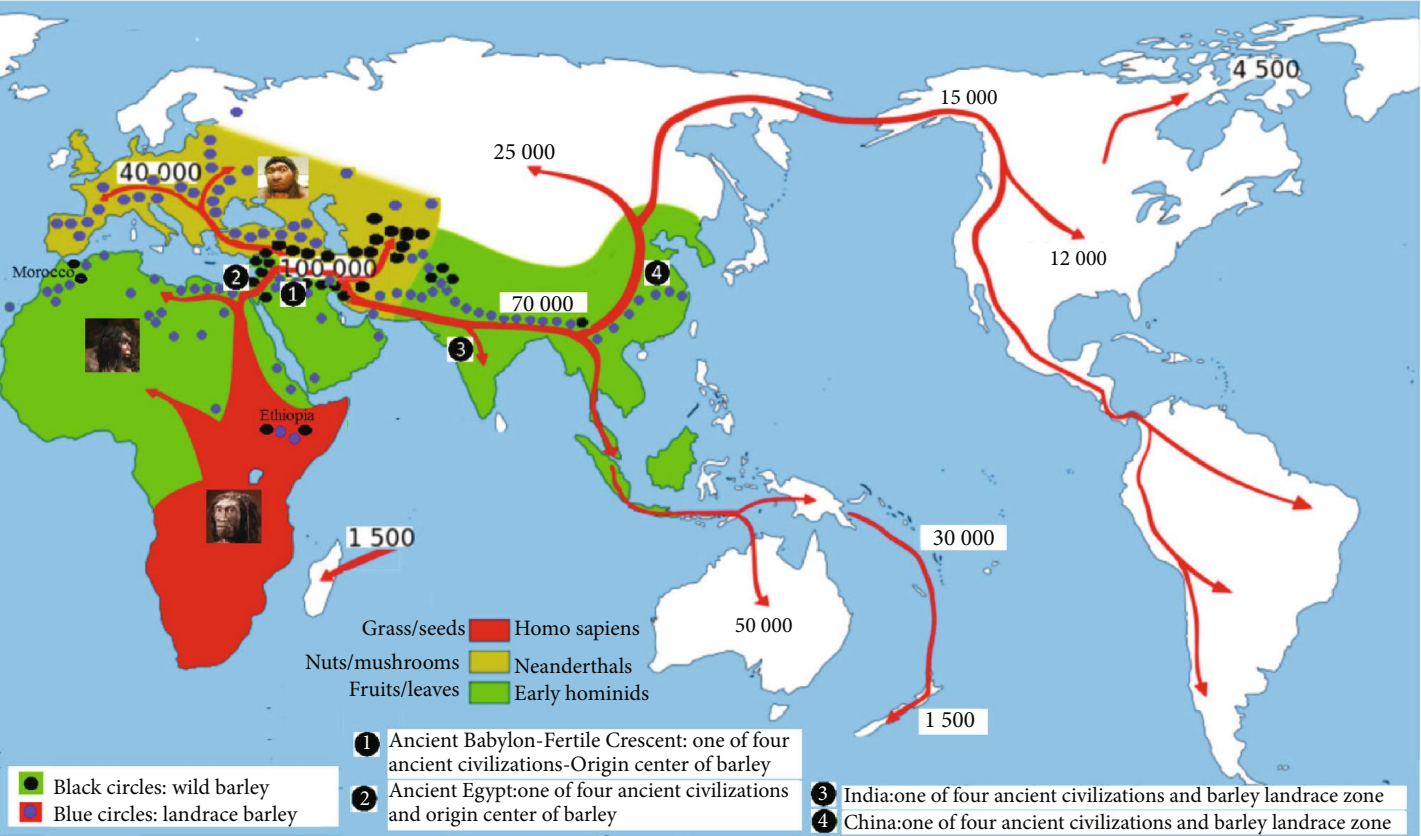
Formation mechanism of functional ingredients in barley associated with preventive chronic diseases and early human migrations according to early human migrations map based on Out of Africa [212] and landrace as well as wild barley zones [214] and so on.

Early hominids used fruits/vegetables and leaves rich in polyphenols and K-Ca as well as vitamins as staple foods to increase the dependence of the human body on these functional ingredients (see Figure 2). Diet played an important role in early hominids evolution [170], but no reports have been delivered to date for diet in coevolution of human chronic diseases. Miocene (23.0~5.3 Ma) apes had a variety of foods that included folivory, soft-fruit eating, and hard-object feeding [170]. The diet of Pliocene (5.3~2.5 Ma) and early Pleistocene (2.5~1.4 Ma) hominids in Africa was mainly fruits and leaves of C3 plants (trees, bushes, shrubs; 5.3~4.1 Ma) which was gradually transformed into grass (C4 plant/tropical grasses and sedges) and hard-object (seeds and nuts) (4.0~1.4 Ma) [170–172], which was due to low availability of fruits in dry and active glacier (1.81~1.55 Ma) as well as migration to warm grasslands. Ethiopia's Pliocene Lucy is one of the oldest and most complete fossils in hominid bones, her death due to fall out vertically and live on tall tree [173]. Early hominids and a*ustralopithecines* inhabited forests and savannas for collinearity found between tasty fruits (fructose/sucrose, quinine, and tannins) and primate sensory perception, which offered evidence of the two-direction evolutionary trend determining taste sensitivity [174]. Our ape ancestors possessed a digestive dehydrogenase enzyme capable of metabolizing ethanol about 10 Ma that they began using fruits fermentation from the forest floor [175]. There were differences in the proportion of meat and vegetables between the early hominids *Australopithecus* and *Paranthropus*; *Paranthropus* ate more hard food than *Australopithecus* [176]. Early hominid *Australopithecus africanus,* like chimpanzees, are dominated by fruit, leaves, and carbon-13-enriched foods about 3 Ma [177]. The worldwide daily consumption of fruits and vegetables as well as tea has become the main tool for prevention of cardiovascular disease, stroke, cancer and diabetes due to their polyphenols modulate tau hyperphosphorylation and beta amyloid aggregation [178]. The anthocyanins and polyphenols for major functional ingredients in blueberry played a key role in preventing 15 chronic diseases [151]. The organopolysulfides and quercetin for major functional ingredients in Allium genus played a key role in preventing 10 chronic diseases [179]. Baobab was cultivated from seeds from 11 countries in East and West Africa, its leaves had the highest vitamin B2 content (1.04 ± 0.05 mg/100 g) from Senegal, adult leaves provided the highest Ca content (3.373%) and young leaves with the highest Ca and K content of Nankoun in Burkina Faso [180]. The diets of early hominids related with five center of crop origin (Mediterranean, Middle East, Central Asia, Indo-Burma, and China-Korea); however, the rich food structure maintained the survival and development of early hominids who lacked survival competition and migration could not improve their intelligence. Therefore, polyphenols and K-Ca as well as vitamins ingredients in fruits/vegetables and leaves as well as grass/seeds (such as Gramineae and its ancestor species of barley) for preventive chronic disease are the results of long-term dependence for diet from Pliocene hominids in Africa to modern human beings (Figure 2).

#### 4.1.2. Formation Mechanism of Depending Functional Ingredients for Neanderthals

Neanderthals used mushrooms and nuts rich in polysaccharide and phytosterols as well as linoleic acid as staple foods to increase the dependence of the human body on these functional ingredients (see Figure 2). Neanderthals as well as early *Homo sapiens* show high dietary variability in Mediterranean evergreen habitats, but less diet in high latitude steppe or coniferous forests [181]. The steppe-like Neanderthals of Belgium feed on the meat of the woolly rhinoceros and wild sheep, while the Neanderthals of the Spanish forest feature root feed on mushrooms and pine nuts [182]; Neanderthals in northern Spain roasted vegetables and used medicinal plants about 2.5~5.0 Ma [183]. Neanderthals ate meat (high chloroprostol) and plants (*5β*-sitosterol) as staple foods [184]. Plant foods of Neanderthals from Iraq and Belgium had the typical modern human diets, which include palms, date, legumes, and grass seeds [185]. 130 medicinal functions (major polysaccharide) in medicinal mushrooms and fungi can prevent and treat more than 10 chronic diseases [186]; however, *β*-glucans in barley preventive 16 chronic diseases. The total polysaccharide content in *Morchella* sp. reached up to 18.4% of dried biomass in a mixture of 1 : 1 of wheat grains and potato peels [187]. *β*-Glucans are group of polysaccharides found in mushrooms, yeasts, seaweed, barley, and oats [13]. Macrofungal *β*-glucans are major *β*-1,3- and *β*-1,6-glycosidic bonds, which have immunomodulatory, anticancer, and antioxidant properties; total *β*-glucan content varied from 13.5% in *A. bisporus* to 40.9% in *T. rutilans* [188]. Phytosterols are diet ingredients found in an array of nuts, seeds, and vegetables which have anticancer activities via their interactions with the plasma cell membrane [189]. Oil palm (*Elaeis guineensis* Jacq.) is one of the highest oil-yield crops in the world; however, palm oil is the largest variety of plant oil produced, consumed (30%), and internationally traded in the world, rich in linoleic acid (10%) that is associated with *egFAD12* gene [190]. 50~55% carbohydrate diets (especially whole-grain breads, vegetables, and nuts) had minimal risk of mortality [191]. Fruits, vegetables, and whole grains were indispensable among four top diets (Mediterranean diet with five best ranked first, DASH diet with lower blood pressure, Flexitarian diet with lose weight and MIND diet with brain health). The diets of Neanderthals was not correlated with the complete center of crop origin, poor diet (meats, mushrooms, and pine nuts) and lack of migration lead to extinction (Figure 2). These results reveal the importance of whole grain and vegetables or fruits in human health; however, lack of three categories of food suggesting the cause of the Neanderthal destruction. Therefore, polysaccharide (*β*-glucan) and phytosterols as well as linoleic acid in mushrooms and nuts as well as palm oil for preventive chronic disease are the results of long-term dependence for diet from Neanderthals in Europe to modern human beings (Figure 2).

#### 4.1.3. Formation Mechanism of Depending Functional Ingredients for Homo Sapiens

*Homo sapiens* not only used grass and seeds rich in GABA and enzymes as well as resistant starch as staple foods to increase the unique dependence of the human body on these functional ingredients (see Figure 2) but also inherited the staple foods of early hominids and Neanderthals. Feeding and diet played key roles in human evolution, especially *Homo sapiens* have a relative masticatory structure similar to that of other primates [192]. *Homo sapiens* moved from Africa into the Middle East about 120 Ka, according to fossils at Skhul and Qafzeh caves in Israel [193]. The aba-miRNA-9497 in belladonna with highly homologous to *homo sapiens* miRNA 378 can target and downregulate human brain-enriched transcription factor (ZNF-691) and gene expression in the human central nervous system [194]. For *homo sapiens,* foragers have greater complexity than farmers or pastoralists; meanwhile, the Old World foragers had significantly higher anisotropy values than New World foragers, but similarity between hard food foragers and hard food farmers [195]. The meat diet abuse by a herbivorous *Homo sapiens* can lead to atherosclerosis [196]. The diets of African *Homo sapiens* associated with center of crop origin in Ethiopia, their migration along eight center of crop origin changed the fate of mankind [9].

Variation in cranial robusticity from 14 geographical *Homo sapiens* associated with cranial shape, size, climate, and neutral genetic distances; however, cranial robusticity may be an adaptation to cold and harsh environments as well as masticatory differences in diet [197], especially higher GABA (such as barley grass 271.3 mg/100 g) grass diets in crop suiting environmental extremes improved intelligence [9]. GABA Contents in picked tea leaves under anoxic treatments at 4 h and 6 h are 16.12 ± 1.05 and 17.13 ± 0.80 mg/kg (fresh weight) [198]. GABA_A_ receptors modulate vigilance, emotions, cognition, and muscle tension, and they are the targets of anxiety-reducing and sedative-hypnotic benzodiazepines and some general anesthetics [199]. *Shisa7* regulates GABA_AR_ trafficking, function, and pharmacology, especially modulates benzodiazepine action in the brain [200].

Barley can be grown in four seasons (spring, summer, autumn, and winter) at 1,900-2,300 m in Yunnan province of China which may be associated with harboring 300 enzymes in barley grass [6]. The diet high in sodium and low four diets (whole grains, fruits, vegetables, nuts, and seeds) were major dietary risk factors for deaths and disability-adjusted life-years globally and in many countries [2]; however, the whole grain is the manifestation of resistant starch type I surrounded by protein matrix and bran layer for making the starch unavailable for enzymes. The least chronic disease of ancient humans due to replacing the salt with seasoning crops, such as onions, ginger, garlic, coriander, pepper, chili, and so on [201]. The coevolution of the preventive human chronic diseases are related to major diets of Vavilov's eight crop origin centers (Ethiopia, Mediterranean, Middle East, Central Asia, Indo-Burma, China-Korea, Mexico-Guatemala, and Peru-Ecuador-Bolivia) [8]. Human chronic diseases are related with six dietary structures (fruits/vegetables, young grass/barley grass, carnivorous, cereals crop, polished rice/wheat flour, and polished rice/wheat+grass powder), but polished rice/wheat+barley grass powder is the most major healthy dietary guidelines for modern humans; therefore, it is necessary to unravel coevolutionary mechanism between preventive chronic diseases and human diet for functional foods [202]. These results support *Homo sapiens* used grass/seeds (rich in GABA and enzymes as well as resistant starch), fruits/vegetables and leaves (rich in polyphenols and K-Ca as well as vitamins), mushrooms, and nuts (rich in polysaccharide and phytosterols as well as linoleic acid) as staple foods to increase the dependence of the human body on these functional ingredients.

### 4.2. Health and Civilization Contribution of Barley Grains and Its Grass

#### 4.2.1. Barley Plays an Important Role in Human Healthy Diet

Barley is the oldest and more important cereal crop with the utmost dietary fiber in the world; its malt, as a functional food, is not only the largest beer raw material in the world but also one of the 300 most commonly used Chinese herbal medicines [6]. Our review point out that barley grass has antidiabetic, anticancer, antidepressant, antioxidant, fatigue, anti-inflammatory, hypolipidemic, antigout, calcium supplementary, and antiacne/detoxifying effects; promotes sleep; regulates blood pressure; enhances immunity; protects liver; reduces hyperuricemia; alleviates atopic dermatitis; improves cognition, constipation, gastrointestinal function; and prevents hypoxia, cardiovascular diseases, and so on [6]. Barley grass powder is known to play a pivotal role in prevention of 20 chronic diseases that involves six molecular mechanism of GABA, flavonoids, SOD, K-Ca, vitamins, and tryptophan [202]; however, barley grains play key roles in prevention of 20 chronic diseases that involves six molecular mechanism of *β*-glucans, polyphenols, arabinoxylan, phytosterols, tocols, and resistant starch.

Modern humans had originated in the progeniture of African *Homo sapiens* with cognitive hominin [203]. The staple foods of modern human are the synthesis of *Homo sapiens* that inherited early hominids and Neanderthals, which carry the Neanderthal DNA due to interbreeding between *Homo sapiens* and Neanderthal took place in the Middle East. Human *Flt3* ligand isolated from transgenic barley seeds is a glycoprotein including *α*(1,3)-fucose and *α*(1,2)-xylose, which showed expression of human growth factor in barley grains with active protein [204]. The peptide LL-37 is a component of the human innate immune system, it accumulated 0.55 mg/kg in the barley grains [205]. Human *fgf-1* gene was fused with barley *α*-amylase signal peptide DNA sequence and expressed in transgenic *Salvia miltiorrhiza* plants driven by 35S promoter; however, recombinant FGF-1 in leaves was 272 ng/ fresh weight [206]. Therefore, functional ingredients in barley grass and grains are essential for the health contribution of modern human (*Homo sapiens*), Neanderthals, and early hominids staple food to prevent and treat human chronic diseases.

#### 4.2.2. Human Health Contribution of Functional Ingredients in Barley

Barley has health beneficial properties and was part of the modern hominid diet; thus, it has evolved in its functional ingredients and contributed to a reduced risk of diseases. It is amazing that barley grains and malt as well as grass powder can prevent or treat more than 20 human chronic diseases. We think the major scientific basis for that is as follows:

First, barley prevent over 20 humans chronic diseases which associated with the similar origin and evolution center of barley and human beings: Ethiopia and Morocco in Africa are top choices for cradle of modern humans *Homo sapiens* and Miocene hominoids as well as are the centers of origin for functional barley (Figure 2) [8, 170, 207]. Ethiopia, Morocco, Fertile Crescent, and Tibet of China have been proposed as centers of barley origin and the primary habitat of wild barley (Figure 2) [207]. Wild barley is a selfing annual grass of predominantly Morocco and Irano-Turanian and Israel-Jordan in arid desert or salt environments, the cold region in Tibet of China and Ethiopia, which has accumulated abundant functional ingredients for drought, salt, and cold resistances. The earliest modern human originate from Ethiopia and Morocco are dated to ~190 Ka and ~315 Ka (Figure 2), respectively [208]. The earliest human occupied high-altitude habitats in the Andes and the Tibetan Plateau, especially Late Pleistocene humans adapted to the severe environments of these glaciated above 4,000-meter elevation in the Bale Mountains of Ethiopia (Figure 2) [209]. Neanderthals in modern-day Iraq and Belgium ate grasses, cooked barley grains, and others.

Second, barley grass powder plays a key role in the promotion of human intelligence in the early stage: the incremental evolution of globular braincase associated with diets of brain health from Ethiopia and Middle East as well as from Mediterranean center of crop origin, especially the highest GABA in crop diets suiting environmental extremes improved intelligence. Brain development is a self-reinforcing process in which brain cells proliferate, differentiate, migrate, and connect functional neural circuit, especially primate-specific features of GABAergic interneuron development [210] on the basis of GABA content in diet. Survival depends on the selection of behaviors adaptive for environment; however, stimulation of dorsal raphe GABA neurons promoted movement in negative but not positive environments to promote environment-specific adaptive behaviors of serotonin [211]. Barley is a major crop in many developed countries [9]; GABA in barley grass suiting environmental extremes (cold, arid, and salt) can significantly increase, which can improve cognition and prevent 12 chronic diseases [6]. The average content of GABA in 31 cultivars that we bred is 271.3 mg/100 g which is 1.8 fold higher than that of other barley crops around the world [9].

Third, healthy effects of functional ingredients of barley grass and grains are the sum of staple foods for early hominids and neanderthals as well as *Homo sapiens*: Early hominids used fruits/vegetables and leaves rich in polyphenols (flavonoids) and K-Ca as well as vitamins (tocols); Neanderthals used mushrooms and nuts rich in polysaccharide (*β*-glucans and arabinoxylan) and phytosterols as well as linoleic acid; *Homo sapiens* used grass and seeds rich in GABA and enzymes (SOD) as well as resistant starch; Modern human used barley grass rich in GABA, flavonoids, SOD, K-Ca, vitamins and tryptophan; however, barley grains rich in *β*-glucans, polyphenols, arabinoxylan, phytosterols, tocols, resistant starch, and so on.

Therefore, barley played an important role in solving the problem of depending functional ingredients of *Homo sapiens* and hominids as well as Neanderthals, especially food safety in the process of migration and evolution from ancient humans to modern people.

#### 4.2.3. Healthy Food Contribution of Barley to Human Migration

Food shortages and survival struggles caused by climate change were the causes of early human evolution of suiting environmental extremes from Africa to Asia and later to Eurasia (Figure 2) [8, 212]. Barley is not only the most widely used cereal crop with comprehensive utilization of forage, materials for intoxicating liquor, functional food, stable food, ornamental weaving, and Chinese medicines but also is the crop with the strongest resistance to stress (drought, cold, and salt) for the highest content of functional components, especially the growth period of barley varies from 70 to 200 days, which can be grown in four seasons in the world or at 1,900~2,300 m in Yunnan province of China. These excellent characteristics become the best food for human migration. Interestingly, there is a striking similarity between the human migration route and barley translocation/evolution route (see Figure 2).

In the Upper Paleolithic Age in Israel, the harvest of barley plants with wild-type brittle spikes occurred at 23 Ka, but the first nonbrittle barley spikes was found on the Fertile Crescent about 10 Ka [213] and on Aswan in Egypt about 17 Ka (Figure 2). Barley has become a founder crop of Neolithic agriculture, especially the close affinity of ancient barley from the Southern Levant and Egypt, consistent with a proposed origin of domesticated barley in the Upper Jordan Valley [214]. The domestication of wild barley in the Fertile Crescent beginning 10 Ka moves into pre-Indus sites (Mehrgarh 9 Ka) and Central Asia between 5.45 and 4.7 Ka and eastern Himalayas by 4.0 Ka; barley arrived on southeastern Tibetan Plateau before the 4.0 Ka cool down, especially millets under cooling climatic conditions are largely replaced by wheat and barley [215]. The spread of farming peoples of Eurasia from the Near East (8.0 Ka), with movements both westward and eastward, especially ancestor of modern South Asians is a mixture between early Holocene populations of Iran and South Asia; however, Yamnaya in the Bronze Age of Europe moved both westward and eastward from north of the Black Sea [216]. Discovery of different crushing apparatuses in mountains of Iran revealed that people were grinding wheat and barley about 11,000 years ago [217]. These results support the healthy food contribution between human migration and barley translocation/evolution, especially climate change increased functional ingredients in barley for preventive chronic diseases.

#### 4.2.4. Contribution of Barley for Promoting World Civilization

This point of view reveals the evolution of human skull morphology on the basis of the hybridization between *H. sapiens* and other hominin species in Morocco at 300,000 years ago, all of which are descendants of the African *Homo sapiens* population [203]. All living people in Europe and Asia carry the same amount of Neanderthal DNA due to the interbreeding between *Homo sapiens* and Neanderthal that took place in the Middle East [193]. Fertile Crescent is the concentrated area of wild barley [214] and the distribution area of ancient Babylonian civilization and ancient Egyptian civilization, among which Jerusalem is the holy place of Judaism, Christianity, and Islam (Figure 2). Barley is one of the oldest crops used by ancient farmers, its cultivation has been optimized by modern humans in the ancient era for less shattering, higher yield and better grains; however, the sharp awn of barley has become an important guarantee for the most resistant birds trouble and large-scale farming civilization in cereal crops.

Climate change stimulated agricultural innovation and exchange across Asia between 5,000 and 1,500 years ago; sorghum and millet made their way from China to Central Asia; wheat and barley moved from Central Asia to the Far East and became a staple food in the north of China at 1.8 Ka, which exchanges across Central and high-altitude Asia coalesced to form the Silk Road (2.114 Ka~1.873 Ka) and Grand Canal for traffic great artery of north-south in ancient China (2.486 Ka) [215]. Tibetan barley (qingke) is derived from eastern domesticated barley, north Pakistan, India, and Nepal between 4,500 and 3,500 years ago, which supports a feral or hybridization origin for Tibetan weedy barley [218]. The rise of barley against stress (drought, cold, salt, and bird) to staple food has increased functional ingredients (especially GABA) in diet to prevent chronic diseases and promoted human civilization. GABA-mediated inhibitory interneurons control memory-encoding CA1 neurons; nucleus incertus (NI) establishes GABAergic inhibitory synapses on interneurons; NI GABAergic cells can regulate hippocampus-dependent episodic memory formation bidirectionally, and its dysfunction may contribute to anxiety-like syndromes [219].

Cities and words and metallurgy as well as complex ceremonial buildings are the four standards of world civilization; Fertile Crescent for one of centers of barley origin is closely related to ancient Babylonian and ancient Egyptian civilizations, especially ancient Babylonian civilizations have the earliest human civilization (agriculture 11 Ka, cities 8~10 Ka, metallurgy 6~7 Ka, words 5.2 Ka, calendar 5 Ka, and systematic religion 4 Ka) in the world; however, ancient Egyptian civilizations has the earliest empire (mathematics 5.2 Ka, geometry 5 Ka, and writing tool 5 Ka). The ancestral blocks of the domesticated barley genomes were descended from all over the Fertile Crescent, especially Levantine (western) and Zagros (eastern) clusters of the origin of agriculture for nine wild barley populations, i.e., Carmel and Galilee, Golan Heights, Hula Valley and Galilee, Judean Desert and Jordan Valley, Lower Mesopotamia, Negev Mountains, North Levant, Sharon, Coastal Plain and Judean Lowlands, and Upper Mesopotamia [220]. The Neolithic crops facilitated the early agricultural establishment; the barley evolution followed the agricultural development in the Near East [213]. For humans from hunter gathering to agriculture about 12 Ka of the Levant in Near East, barley was a founder crop for converting the brittle floral axis of the wild-type into a tough and nonbrittle spike, which made a major contribution to the emergence of early agrarian societies [221]. For ancient Indian civilization, the earliest known farming cultures in south Asia emerged in the hills of Balochistan in Pakistan about 9.2 Ka; however, seminomadic peoples domesticated wheat, barley, sheep, goat, and cattle [217]. For ancient Chinese civilization, the earliest beer recipe in China included broomcorn, millet, barley, Job's tears, and tubers around 5,000 y ago, which may have motivated the initial translocation of barley from the Western Eurasia into the Central Plain of China [222].

### 4.3. Action Mechanisms for Barley to Combat Chronic Diseases

Western medicine solves the issue of human organs, and traditional Chinese medicine solves the issue of human body system; however, functional food is to address the problem of human cells. Barley grains and its grass not only are the best functional food that provides nutrition and eliminates toxins from cells in human beings, which are rich in 30 ingredients to combat more than 20 chronic diseases but also have all the nutrients needed for cell nutrition and detoxification, which is the result of the long-term coevolution of the dietary structure of ancient apes for plants and early *Homo sapiens* with the staple food of barley. Interestingly, the types of prevention and treatment of human chronic diseases by key functional components in barley grain were in order: *β*-glucans (16)>polyphenols (13)>arabinoxylan (7) = tocols (7)>phytosterols (5)>resistant starch (4), but GABA (13)>flavonoids (11)>SOD (8)>K-Ca (7) = vitamins (7)>tryptophan (3) in barley grains. The 12 key functional components of barley grains and its grass not only play an important role in the prevention and treatment of more than 20 chronic human diseases but also interact with other 30 nutritional functional components to provide the possibility to solve hundreds of human diseases caused by cell undernutrition and their detoxification disorders. The unique theory and practice system of the whole regulation improve the immunity of Chinese medicine to prevent and treat human diseases. With advances in science and technology, barley will also find many behavioral mechanisms that combat human diseases, such as barley malt has been used in many prescriptions for the prevention and treatment of COVID-19 in lots of provinces in China, which includes Jiangxi, Guangdong, Gansu, Guizhou, and Jiangsu province in China [223]. Dietary restriction is an activator of prolongevity molecular pathways based on an escape from costs incurred under nutrient-rich conditions [224]. Chronic disease deaths due to heredity, environment, and lifestyle are as high as 41 million, accounting for more than 70% of all deaths worldwide [225]. These results support that human has only new cell disease theory, which are made up of sixty million cells, more than 1,000 diseases are due to cell nutritional deficiencies and detoxification disorder caused by the disease [226].

## 5. Countermeasures for Increasing Functional Ingredients of Barley

### 5.1. Exploration of Excellent Germplasm with High Functional Ingredients in Barley

The variation of grain functional ingredients in different genotypes of barley was very larger, such as *β*-glucan (2.40~11.00%), resistant starch (0.2~24.0%), arabinoxylan (0.70~2.13%), polyphenols (150~300 mg/100 g), phenolic acids (336.29~453.94 mg/100 g), total flavones (37.93~236.91 mg/100 g), total alkaloids (6.36~44.63 mg/100 g), total anthocyanin (4.9~103.7 mg/100 g), total tocols (0.85~12.49 mg/100 g), GABA (0.10~30.67 mg/100 g), folates (51.8~103.3 mg/100 g), phytosterols (76.1~115.3 mg/ 100 g), Ca (6.84~115.00 mg/100 g), and Fe (0.88~15.61 mg/100 g) (see Table 1). The variation of grass functional ingredients in different genotypes of barley was very larger, such as vitamin A (14.4~25.0 mg/100 g), vitamin B3 (2.20~16.49 mg/100 g), vitamin C (19.4~548.0 mg/100 g), vitamin E (6.1~46.1 mg/100 g), Ca (330~819 mg/100 g), K (2400~4300 mg/100 g), superoxide dismutase (416~1382 U/g), catalase (675~935 U/g), lutonarin (200.0~540.0 mg/100 g), saponarin (300.0~1260.0 mg/100 g), total polyphenol (1.03~1.08%), total flavonoid (487.5~593.4 mg/100 g), GABA (125~183 mg/100 g), and tryptophan (290~1400 mg/100 g) [6]. These are the results of variation of functional ingredients in grains and grass powder of less than 1,000 cultivated barley varieties. Genetic structure of 22,621 accessions of wild and domesticated barley in the genebank based on 171,263 SNP markers insights into the global population structure and redundancies and coverage gaps of domesticated barley [207]; However, 18,773 barley germplasm resources (2,585 wild barley) were preserved in the Chinese Crop Genebank. The detection of functional ingredients in grains and grass powder of more than 10,000 accessions barley gremplasm especially combinated with high-density SNP markers can not only reveal the molecular mechanism of functional ingredients and their molecular breeding and gene editing techniques but also make great contributions for improving human health by increasing functional ingredients in barley grains and grass powder.

### 5.2. Breeding Excellent Cultivars with High Functional Ingredients in Barley

Some excellent barley varieties have very high functional ingredients both in grains and grass powder, which are the result of adaptation to high and low temperature, drought and waterlogging, long and short sunshine, and day and night changes caused by altitude, latitude, and seasonal differences.

First of all is the breeding of high functional ingredients in barley grains. We have bred some functional barley by crossbreeding between the highest functional ingredients germplasm (*β*-glucan >8.5%, resistant starch >10%, arabinoxylan >2%, or polyphenols >0.2%) and cultivated barley at low temperature and drought as well as high altitude, such as Yunke 1 and Zangqing 25 with high *β*-glucan, Yungongmai 1, and Yungongmai 2 with high vitamin C.

Secondly is the breeding of high functional ingredients in barley grass. We have bred some functional barley by crossbreeding between the highest functional ingredients germplasm (GABA >0.2%, K >3.0%) and cultivated barley at low temperature and drought as well as high altitude. The average content of GABA in 31 cultivars that we bred is 271.3 mg/100 g which is 1.8 fold higher than that of other barley crops around the world, especially barley grass powder of Yungong brand contains 62 times GABA (327.5 mg/100 g) and 99 times Ca as well as 31 times K than those of polished rice [9].

In addition, the functional ingredients of barley grain and grass powder can also be greatly improved by gene editing technology, which needs to be further studied and enriched in the future.

### 5.3. Optimization of Ecological Conditions for High Functional Ingredients of Barley

Functional ingredients of barley grain and its grass powder have larger variation due to latitude, altitude, season, light, temperature, water, day, and night. All the high-altitude (1,200~3,500 m) hulless barley can increase higher functional ingredient content than that of plains (97~126 m altitude) [25]. Therefore, it is necessary to promote the functional components of the barley grain and its grass powder under the best ecological conditions, and we also make a useful exploration of the functional components at different altitudes and in different seasons. In addition, the functional ingredients of different parts of barley grain (see Table 1) and the functional ingredients of different grass cutting stages were different.

## 6. Conclusion and Future Perspectives

Barley is the oldest and the richest functional food among global cereals. Its grains are rich in *β*-glucan; polyphenols (phenolic acids, flavonoids, and anthocyanins), polysaccharide (arabinoxylan), phytosterols (*β*-sitosterol, campesterol), tocols (*β*-tocotrienol, *α*-tocotrienol, *β*-tocopherol, *α*-tocopherol), resistant starch, alkaloid, GABA, folates, linoleic acid, phytate, and so on. This review paper summarizes the obvious efficacy of barley grains that includes antidiabetes, antiobesity, anticancer, antioxidants, anti-inflammation, immunomodulation, cardioprotection, gastroprotection, and hepatoprotection properties, and also, barley grains can lower blood pressure; prevent cardiovascular diseases; optimize cholesterol; improve bowel health and metabolic syndrome; prevent heart disease; reduce chronic kidney disease; decrease stroke; alleviate allergic rhinitis and atopic dermatitis; and accelerate wound healing activities.

Barley grains, grass, straw, husk, bran, and fine powder are rich in 30 ingredients and food structure to defeat chronic diseases during human migration, especially molecular mechanisms of six functional ingredients barley grass (GABA, flavonoids, SOD, K-Ca, vitamins, and tryptophan) and grains (*β*-glucans, polyphenols, arabinoxylan, phytosterols, tocols, and resistant starch) involve to combat more than 20 chronic diseases. These results suggest that barley plays an important role in a healthy diet and in the promotion of early human intelligence. In particular, the healthy effects of functional components of barley grains and grass are the result of long-term continuous evolution of early hominids (fruits/vegetables and leaves rich in polyphenols, K-Ca, and vitamins), Neanderthals (mushrooms and nuts rich in polysaccharides, phytosterols, and linoleic acids), and *Homo sapiens* (grasses and seeds rich in GABA, enzymes, and resistant starch), which associate with modern humans originating in the progenitor of African *Homo sapiens* with cognitive hominin, especially after interbreeding between *Homo sapiens* and Neanderthals that took place in the Middle East. The migration route from Africa to Asia and then to Eurasia is basically consistent with the origin and spread of barley and its domestication path, which indirectly supports that barley against stress (drought, cold, and salt) enriched with functional ingredients prevented chronic disease from ancient humans to modern people. Fertile Crescent is the concentrated area of wild barley and the distribution area of ancient Babylonian civilization and ancient Egyptian civilization, among which Jerusalem is the holy place of Judaism, Christianity, and Islam. These results indirectly support this great contribution of barley for promoting world civilization.

The polyphenols in fruits/leaves and polysaccharide in mushrooms/nuts as well as GABA in grass/seeds for prevention of chronic disease are associated with depending functional ingredients for diet from Pliocene hominids in Africa to modern humans. Ethiopia and Morocco in Africa are top choices for cradle of modern humans *Homo sapiens* and Miocene *hominoids* as well as are the centers of origin for functional barley. Food shortages and survival struggles caused by climate change were the causes of early human evolution associated with GABA in the barley grass increased sharply under environmental extremes from Africa to Asia and later to Eurasia, especially GABA in crop diets suiting environmental extremes improved intelligence. These results support findings that barley and its grass may be the best functional food crop, especially barley prevents over 20 human chronic diseases based on six functional ingredients of barley grass and grains due to three aspects of the scientific basis, i.e., the similar origin and evolution center of barley and human, the promotion of human intelligence in the early stage, and the sum of staple foods for early hominids and Neanderthals as well as *Homo sapiens*.

We put forward the strategy of increasing the functional ingredients of barley grain and its grass powder that is as follows: (1) exploration of excellent germplasm with high functional ingredients in barley; (2) breeding excellent cultivars with high functional ingredients in barley; (3) optimization of ecological conditions for high functional ingredients of barley. In addition, the functional ingredients of different parts of barley grain and the functional ingredients of different grass cutting stages are different.

Although therapeutic mechanisms of functional ingredients in barley grains and grass powder for prevention of human chronic diseases seem a very complicated task, and functional food for therapeutic interventions opens up new ways, it is necessary to find further scientific evidence that demonstrates the health effects of functional ingredients of barley and their extracts from barley on the treatment of chronic diseases. Barley is one of the most exciting potential natural sources for the development of functional foods and new drugs with improved efficiency and safety. Although we have found some relationship of origin and migration between human and barley, especially preventive role of barley for chronic diseases of human beings, it is necessary to conduct more systemic studies to unravel coevolutionary interconnection mechanism between chronic diseases prevention and human diet for barley functional foods. Unfortunately, so far there, is no evidence provided of barley evolving as part of evolutionary consumption. Barley plays an important role in promoting the development of functional food and has a potential underlying molecular mechanism and formation as well as action mechanism, which is worthy of further study. This review can be used as a starting point for novel nutraceuticals and functional foods and drugs for barley to improve the prognosis of chronic diseases.

## Figures and Tables

**Table 1:** Functional and nutrient compositions of barley grains.

Composition	Kernel position	Mean ± SD	Range	References
*β*-glucan (%)	Whole grains	4.61 ± 0.45	2.40~11.00	[21, 29, 39]

Resistant starch (%)	Whole grains	3.63 ± 2.32	0.2~24.0	[73–75]

Arabinoxylan (%)	Endosperms	0.67 ± 0.06	0.53~0.90	[55]
	Barley bran	4.66 ± 3.35	1.97~8.42	[29, 30]
	Grains flour	1.31 ± 0.73	0.70~2.13	[29, 30]

Polyphenols (mg/100 g)	Whole grains	231.61 ± 34.26	150.0~300.0	[37, 118]
	Barley bran	421.84 ± 24.46	376.1~443.5	[30]
	Grains flour	140.41 ± 10.21	129.9~160.7	[30]

Phenolic acids (mg/100 g)	Whole grains	414.70 ± 32.86	336.29~453.94	[39]

Total flavones (mg/100 g)	Whole grains	80.64 ± 17.15	37.93~236.91	[37, 39, 75]

Flavonoids (mg/100 g)	Whole grains	12.51 ± 10.14	6.20~30.08	[18]

Catechin (mg/100 g)	Whole grains	2.25 ± 0.94	0.90~4.27	[18, 227]

Quercetin (mg/100 g)	Purple grains	3.51 ± 2.24	2.00~6.08	[18, 227]

Kaempferol (mg/100 g)	Whole grains	3.66 ± 14.87	1.27~6.31	[18, 227]

Myricetin (mg/100 g)	Whole grains	11.07 ± 22.25	0~73.30	[227]

Total alkaloid (mg/100 g)	Whole grains	25.96 ± 1.41	6.36~44.63	[228]

Total anthocyanin (mg/100 g)	Whole grains	35.50 ± 23.82	4.9~103.7	[229]
	Barley bran	256.05 ± 137.67	158~353.4	[54]
	Refined flours	39.15 ± 25.67	21.0~57.3	[54]

Proanthocyanidin (mg/100 g)	Whole grains	6.97 ± 3.84	1.58~13.18	[18]

Total tocols (mg/100 g)	Whole grains	5.85 ± 3.51	0.85~12.49	[21, 66, 70, 71]

Antioxidant activity (%)	Whole grains	41.55 ± 7.82	24.10~82.00	[37]

GABA (mg/100 g)	Whole grains	8.00 ± 3.92	0.10~30.67	[75]

Protein %	Whole grains	14.92 ± 0.13	9.51~20.46	[135]

Folates (mg/100 g)	Whole grains	71.24 ± 16.62	51.8~103.3	[18, 23]

Phytosterols (mg/100 g)	Whole grains	91.13 ± 21.14	76.1~115.3	[18]

P (mg/kg)	Whole grains	2,592.9 ± 1,045.5	936~6538	[24]

K (mg/kg)	Whole grains	4,801.7 ± 1,839.2	207~9162	[24]

Ca (mg/kg)	Whole grains	568.3 ± 235.1	68.4~1150.0	[24]

Mg (mg/kg)	Whole grains	1,249.8 ± 392.7	308.4~2164.0	[24]

Fe (mg/kg)	Whole grains	52.7 ± 31.3	8.8~156.1	[24]

Zn (mg/kg)	Whole grains	39.5 ± 15.5	9.4~76.2	[24]

Cu (mg/kg)	Whole grains	14.1 ± 10.3	0.6~68.0	[24]

Mn (mg/kg)	Whole grains	29.3 ± 24.8	5.8~120.0	[24]

Na (mg/kg)	Whole grains	190.5 ± 104.7	6.7~611.5	[24]

S (mg/kg)	Whole grains	1,505.2 ± 262.8	686.0~2363.5	[24]

ABTS-IR50 (g/L)	Grain alkaline extract polysaccharide	2.12 ± 0.35	1.74~2.84	[17]
ABTS-TEAC (mg/g)		8.94 ± 1.34	6.50~10.61	[17]
FRAP (*μ*mol/g)		90.58 ± 21.61	51.1~131.1	[17]
ORAC (*μ*mol/g)		380.28 ± 161.24	147.81~652.46	[17]

ABTS-IR50 (g/L)	Grain water extract polysaccharide	10.59 ± 1.69	7.41~13.43	[17]
ABTS-TEAC (mg/g)		1.79 ± 0.31	1.37~2.49	[17]
FRAP (*μ*mol/g)		32.14 ± 9.35	15.80~41.80	[17]
ORAC (*μ*mol/g)		206.49 ± 106.83	71.49~396.57	[17]

ABTS = 2,2-azino-bis(3-ethylbenzothiazoline-6-sulfonic acid) diammonium salt; ORAC = oxygen radical absorbance capacity; TEAC = Trolox equivalent antioxidant capacity; FRAP = Ferric reducing antioxidant power.

**Table 2:** Functional ingredients of barley grass and grains for similar preventive chronic disease.

Preventive chronic disease	Functional ingredients in grass [6]	Functional ingredients in grains	References in grains
Antidiabetes	Saponarin; dietary fibre, Ca; AMP-activated protein kinase, polyamines; GABA; SOD	*β*-glucan; phenolic compounds; polysaccharide; tocols; phytosterols, resistant starch	[18, 83, 84, 129, 165]
Hypolipidemic effects or antiobesity	Saponarin; *α*-tocopherol; 2^″^-O-glycosyl isovitexin, polysaccharide	*β*-glucan, resistant starch, tocols, dietary fiber, polyphenols, polysaccharide, phytosterols	[18, 36, 91, 92, 94–96, 163]
Anticancer	Alkaline, flavonoids, chlorophyll; tricin; SOD	*β*-glucan, phenolics, arabinoxylan, phytosterols, lignan, resistant starch	[62, 100, 102, 103, 111, 155]
Antioxidation	Chlorophyll; lutonarin, saponarin; isoorientin, orientin; *γ*-tocopherol, glutathione; SOD, flavonoid, GABA	Polyphenols, phenolics, anthocyanin, VE, tocotrienol, polysaccharide, GABA	[17, 33, 38, 54, 105–107, 150]
Anti-inflammation	Chlorophyll; saponarin; SOD; GABA; tryptophan	*β*-Glucans, vanillic acid, lignans, arabinoxylan	[110–113]
Immunomodulation	Arabinoxylan; polysaccharide; GABA	*β*-Glucans, arabinoxylan	[114, 115]
Cardioprotection	K, GABA	*β*-d-glucan	[116]
Blood pressure regulation	Saponarin; lutonarin, K, Ca; GABA	*β*-Glucans	[98, 121]
Bowel health	Dietary fiber	*β*-Glucans, dietary fiber	[122, 123]
Improve gastrointestinal	Dietary fiber; selenium; GABA	*β*-Glucans	[122]
Hepatoprotection	Saponarin; SOD; GABA,	*β*-Glucans, phenolics, pentosan	[118, 129, 130]
Cardiovascular disease prevention	Saponarin; tryptophan; vitamins (A,B1, C,E), SOD; K, Ca; GABA	*β*-Glucans, arabinoxylan, polyphenols, phytosterols, lignans, tocols, folate	[17, 18, 98]
Atopic dermatitis alleviation	GABA, SOD	GABA, extract P	[138, 139]
Antiaging	Excavate functional components	Excavate functional components	[18]

**Table 3:** Different functional ingredients of barley grass and grains for discrepant preventive chronic disease.

Barley grass		Barley grains		
Preventive role	Functional Ingredients [6]	Preventive role	Functional Ingredients	References
Improve cognition	GABA, K, SOD	Hypocholesterol	*β*-glucans	[119]
Beauty antiacne/detox	Metallothioneins	Reduce chronic kidney disease	*β*-glucans	[90]
Antigout/ hyperuricemia	Alkaloid, SOD	Improve metabolic syndrome	*β*-glucans	[127]
Calcium supplement	Ca	Wound healing acceleration	*β*-glucans	[132, 133]
Antihypoxia/antifatigue	Flavones (lutonarin and saponarin)	Heart failure prevention	*β*-d-glucan, phenolics, tocols, linoleic acid, folate	[18, 123, 136]
Antidepressant	GABA; saponarin; vitamins; minerals	Stroke prevention	Low protein, extract P	[140, 141]
Promote sleep	GABA, Ca, K, tryptophan, vitamin C	Allergic rhinitis alleviation	Fermented barley extract	[138]
Lustihood	Excavate functional components	Body strength	Excavate functional components	[18]
Bone injury recovery	Excavate functional components	Prevent cholelithiasis	Excavate functional components	[18]
